# Stimulation of extrinsic sympathetic nerves differentially affects neurogenic motor activity in guinea pig distal colon

**DOI:** 10.14814/phy2.15567

**Published:** 2023-01-12

**Authors:** David J. Smolilo, Timothy J. Hibberd, Marcello Costa, Phil G. Dinning, Lauren J. Keightley, Dayan De Fontgalland, David A. Wattchow, Nick J. Spencer

**Affiliations:** ^1^ College of Medicine and Public Health Flinders University Adelaide South Australia Australia; ^2^ Department of Surgery Flinders Medical Centre Bedford Park South Australia Australia; ^3^ Department of Gastroenterology Flinders Medical Centre Bedford Park South Australia Australia

## Abstract

The speed of pellet propulsion through the isolated guinea pig distal colon in vitro significantly exceeds in vivo measurements, suggesting a role for inhibitory mechanisms from sources outside the gut. The aim of this study was to investigate the effects of sympathetic nerve stimulation on three different neurogenic motor behaviors of the distal colon: transient neural events (TNEs), colonic motor complexes (CMCs), and pellet propulsion. To do this, segments of guinea pig distal colon with intact connections to the inferior mesenteric ganglion (IMG) were set up in organ baths allowing for simultaneous extracellular suction electrode recordings from smooth muscle, video recordings for diameter mapping, and intraluminal manometry. Electrical stimulation (1–20 Hz) of colonic nerves surrounding the inferior mesenteric artery caused a statistically significant, frequency‐dependent inhibition of TNEs, as well as single pellet propulsion, from frequencies of 5 Hz and greater. Significant inhibition of CMCs required stimulation frequencies of 10 Hz and greater. Phentolamine (3.6 μM) abolished effects of colonic nerve stimulation, consistent with a sympathetic noradrenergic mechanism. Sympathetic inhibition was constrained to regions with intact extrinsic nerve pathways, allowing normal motor behaviors to continue without modulation in adjacent extrinsically denervated regions of the same colonic segments. The results demonstrate differential sensitivities to sympathetic input among distinct neurogenic motor behaviors of the colon. Together with findings indicating CMCs activate colo‐colonic sympathetic reflexes through the IMG, these results raise the possibility that CMCs may paradoxically facilitate suppression of pellet movement in vivo, through peripheral sympathetic reflex circuits.

## Graphical abstract

Colonic motor complexes (CMCs) activate gut‐projecting sympathetic prevertebral neurons, but CMCs are one of several neurogenic motor behaviors that contribute to colonic motility. We tested the sensitivity of CMCs, transient neural events (TNEs), and pellet propulsion to sympathetic nerve stimulation. TNEs and pellet propulsion were inhibited at lower stimulation frequencies than CMCs. The findings raise the possibility that CMC‐evoked sympathetic reflexes may exert inhibitory effects on pellet propulsion without self‐extinguishing.

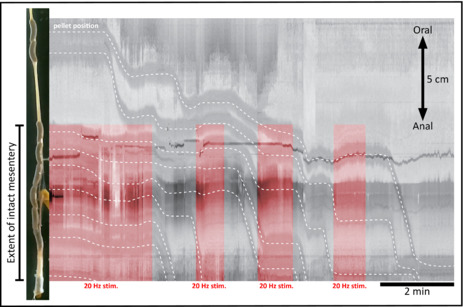

## 1 INTRODUCTION

The enteric nervous system (ENS) embedded within the gut wall contains neural circuits for controlling most gut functions (Furness, 2006) within two main ganglionated plexuses: the myenteric plexus, principally responsible for controlling gut motility (Costa & Furness, 1976), and the submucosal plexus which contributes to control of mucosal secretion (Frieling et al., 1992; Weber et al., 2001) and vascular tone (Vanner et al., 1993; Vanner & Surprenant, 1996). The presence of complete neural circuits makes the gut capable of basic motor functions even when isolated from the central nervous system. Indeed, a diverse range of ENS‐driven motor behaviors have been identified in isolated guinea pig colon, including colonic motor complexes (CMCs), transient neural events (TNEs), distal and proximal colonic migrating motor complexes (DCMMCs/PCMMCs), and peristalsis/pellet propulsion (Costa et al., 2015, 2021; Costa, Keightley, Wiklendt, Hibberd, Arkwright, Omari, Wattchow, Brookes, et al., 2019; Costa, Keightley, Wiklendt, Hibberd, Arkwright, Omari, Wattchow, Zagorodnyuk, et al., 2019; D'Antona et al., 2001; Hennig et al., 2010; Smith et al., 2003).

However, gut autonomy is tempered by extrinsic sympathetic and parasympathetic inputs in vivo, which have powerful (and usually opposing) modulatory effects on gut function, including gut motility. Sympathetic input reduces colonic smooth muscle contractility (Garry & Gillespie, 1955; Gillespie, 1962; Mackenna & McKirdy, 1972). Distension‐evoked phasic contractions and the speed of pellet propulsion in guinea pig distal colon can be significantly reduced by sympathetic input via an α_2_ receptor‐dependent mechanism (Crema et al., 1970) (Gribovskaja‐Rupp et al., 2014), likely to involve inhibition of both ascending and descending cholinergic neurotransmission in the myenteric plexus (Spencer et al., 1999).

Interestingly, the rate of emptying of the endogenous pellets from guinea pig distal colon in vitro (Costa, Keightley, Wiklendt, Hibberd, Arkwright, Omari, Wattchow, Zagorodnyuk, et al., 2019) far exceeds physiological pellet expulsion rates in vivo (Costa et al., 2021; Elfers et al., 2021). Moreover, the distal colon is usually full of natural pellets (Costa et al., 2021). This raises the possibility that sympathetic inhibition in vivo slows colonic emptying.

Sympathetic reflexes represent a potential mechanism that may contribute to pellet retention. Two such reflex pathways can inhibit gut motility (Furness & Costa, 1974b). One is the central intestino‐intestinal inhibitory reflex (Furness & Costa, 1974a), involving spinal sensory nerves that activate, via spinal interneurons, sympathetic preganglionic, and postganglionic neurons to the gut. This central reflex is regarded a defense mechanism to gut inflammation or irritation, causing generalized cessation of gut movements (Wattchow et al., 2021). The second type is the peripheral intestino‐intestinal pathway, involving enteric viscerofugal neurons whose axons project outside the gut wall, synapsing on gut‐projecting postganglionic sympathetic neurons (Furness & Costa, 1974b; Kuntz, 1940; Kuntz & Saccomanno, 1944; Szurszewski & Miller, 1994). The physiological role of this peripheral reflex pathway remains unclear. Recent experiments revealed synaptic activation of viscerofugal neurons during CMCs activates the peripheral reflex pathway, co‐activating postganglionic sympathetic neurons to the same colonic region (Hibberd et al., 2020).

Thus, CMCs could inhibit pellet propulsion via peripheral sympathetic reflexes. However, this possibility would require a differential sensitivity of CMCs compared to other neurogenic motor behaviors to sympathetic activity. The aim of this study was therefore to examine the effects of sympathetic nerve stimulation on different neurogenic colonic motility patterns; specifically, whether CMCs were less sensitive to sympathetic inhibition than either pellet propulsion or “transient neural events” (TNEs).

## 2 METHODS

### 2.1 Animal handling and tissue collection

**FIGURE 1:**
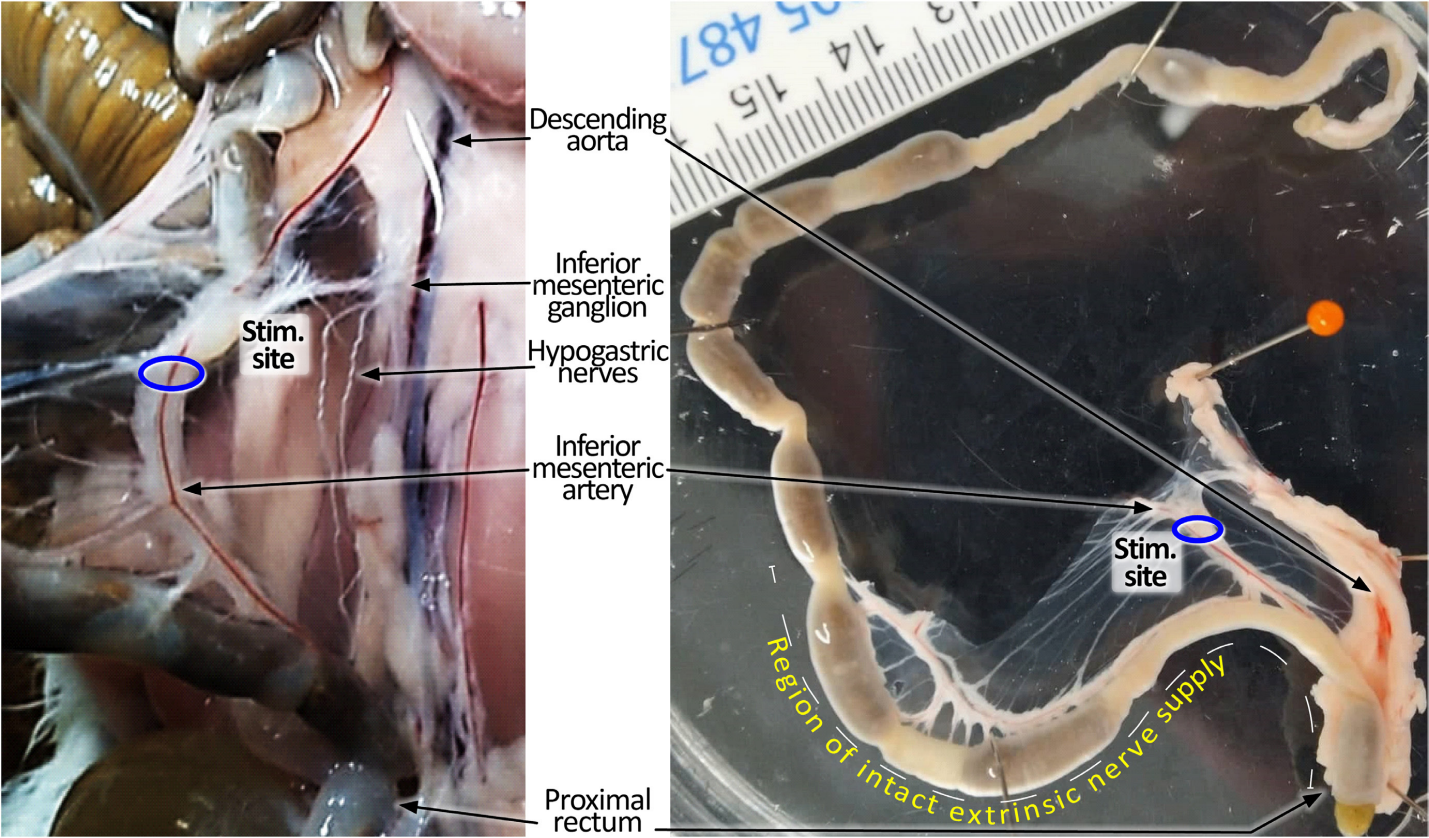
Anatomy of guinea pig surrounding the stimulation site along the inferior mesenteric artery (IMA). The panel on the left is a photograph demonstrating the mesentery of the distal part of the distal colon, in vivo. The panel on the right shows the dissected specimen from the same animal before it is placed into the organ bath, in vitro. Note the distal segment of the preparation has intact extrinsic nerve connections to the stimulation site at the IMA, but the mesentery and extrinsic nerves in the proximal segment were severed close to the gut wall.

All experimental procedures were approved by the Animal Welfare Committee of Flinders University (Projects 844/12, 908/12, 916/12). Duncan‐Hartley guinea pigs of either sex, within a weight range of 380–500 g, were killed by isoflurane inhalation followed by exsanguination. The abdominal cavity was entered through a midline skin incision. The distal colon with mesentery and inferior mesenteric ganglion intact were removed and placed in a dissection dish containing Krebs solution at room temperature (mM: 118 NaCl, 4.7 KCl, 1.0 NaH_2_PO_4_, 25 NaHCO_3_, 1.2 MgCl_2_, 11 D‐glucose, and 2.5 CaCl_2_) carbogenated with 95% O_2_–5% CO_2_ (Figure 1). In experiments on empty colon, fecal pellets were allowed to evacuate spontaneously or were gently flushed with Krebs solution. In three experiments, natural pellet expulsion was prevented by a rubber loop placed at the anal end of the isolated segment which was removed just prior to recording. Preparations were placed in organ baths containing carbogenated Krebs solution (400 ml), maintained at 36.5°C. Several experimental methods were used individually or in combination to record diameter maps (video), electrical activity of smooth muscle (suction electrodes), and intraluminal pressure changes (manometry catheters).

### 2.2 Electrical stimulation of extrinsic nerves associated with the inferior mesenteric artery

Electrical stimuli were generated with a Grass SD9 stimulator (Grass Instruments) and delivered via platinum ring electrode located around the inferior mesenteric artery (IMA) and the colonic nerves that run with it (collectively referred in this paper as the IMA pedicle), just distal to the IMG (Figure 1). It is important to note that approximately half the length of distal colon preparations (the most distal half) had intact extrinsic nerve pathways arising from the IMA pedicle. This is indicated by the dashed line adjacent to the photograph of the ex vivo preparation in Figure 1. During TNE, CMC, and pellet propulsion recordings, the effects of electrical stimulation of sympathetic nerves in the IMA pedicle were limited by the extent of the intact extrinsic nerve supply. For IMA pedicle stimulation, the neurovascular trunk was held within the ring electrode using a silk tie, which also allowed it to be removed and re‐inserted during the experiment. The standard stimulus parameters were 50 V and 0.5 ms pulse width, applied at frequencies of 1, 2, 5, 10, and 20 Hz. The duration of stimulus trains was timed manually. A minimum 1 min interval was allowed between stimulus trains during TNE recordings; more than 3 min was allowed during CMC recordings and single pellet propulsion studies.

### 2.3 Extracellular electrophysiological recordings of TNEs

For TNE recordings, two Krebs‐filled glass suction electrodes (AgCl, 250 μm) with heat‐polished glass tips (0.86 mm ID, 1.5 mm OD) were applied to the colonic serosa; one located within the region of intact extrinsic nerve supply, and the other outside. Voltage was amplified by AC‐coupled amplifier (ISO‐80; WPI, Sarasota, FL) and a DC‐coupled amplifier (DAM‐50, Sarasota, FL) and recorded at 1 kHz (PowerLab 16/35, LabChart 7; ADInstruments, BellaVista, NSW, Australia). The ISO‐80 recording was primarily used to visualize fast action potentials, with low‐pass and high‐pass filters set at 100 Hz and 5 Hz, respectively. The DAM‐50, used in DC mode, was useful in demonstrating events such as excitatory / inhibitory junction potentials (EJP/IJP) and slow waves (Costa et al., 2021). Recordings were analyzed using LabChart 7 (ADInstruments).

### 2.4 Diameter maps (DMaps) of pellet propulsion

As previously described (Costa et al., 2013; Dinning et al., 2012), gut movements were recorded by digital video camera (Logitech Carl Zeiss Tessar 1080 Webcam) and LabChart 7 Pro software (ADInstruments). Spatiotemporal maps of changes in gut diameter were made using custom‐written software in Matlab (MathWorks, Natick, MA) and PlotHMR written in Java (Sun Microsystems, Santa Clara, CA) (Dinning et al., 2014). Maximal dilatation of the gut on the Dmap (maximal diameter) is represented by black pixels, while regions of maximal contraction (minimal diameter) appear white.

### 2.5 Intraluminal manometric recordings of CMCs

Intraluminal pressure was recorded using a solid‐state manometry catheter of 4.15 mm outer diameter, incorporating 36 sensors spaced at 1 cm intervals (Given Imaging, Los Angeles, CA). The catheter was inserted from the oral end of preparations. Cotton suture at both ends of preparations held the gut length and position constant, relative to the catheter. The catheter was initially calibrated in millimeters of mercury and data acquisition was at 50 Hz using Sandhill Scientific software (Insight acquisition system, Denver, CO). Analysis was performed using PlotHRM. The 4.15 mm diameter catheter itself provides a distending stimulus that evokes ongoing CMCs in guinea pig distal colon (Costa, Keightley, Wiklendt, Hibberd, Arkwright, Omari, Wattchow, Brookes, et al., 2019).

Statistical analysis was performed on preparation averages by ANOVA (one‐way or two‐way, repeated measures), Student's two‐tailed *t*‐test for paired data, or Chi‐squared test using Prism 8 (GraphPad Software, Inc. La Jolla, CA, USA). Statistical differences were considered significant if *p* < 0.05. Methods for pairwise comparisons following ANOVA are described for each instance in text. Data are presented as mean ± standard deviation. Lower case “*n*” indicates the number of animals.

## 3 RESULTS

### 3.1 Transient neural events (TNEs)

**FIGURE 2:**
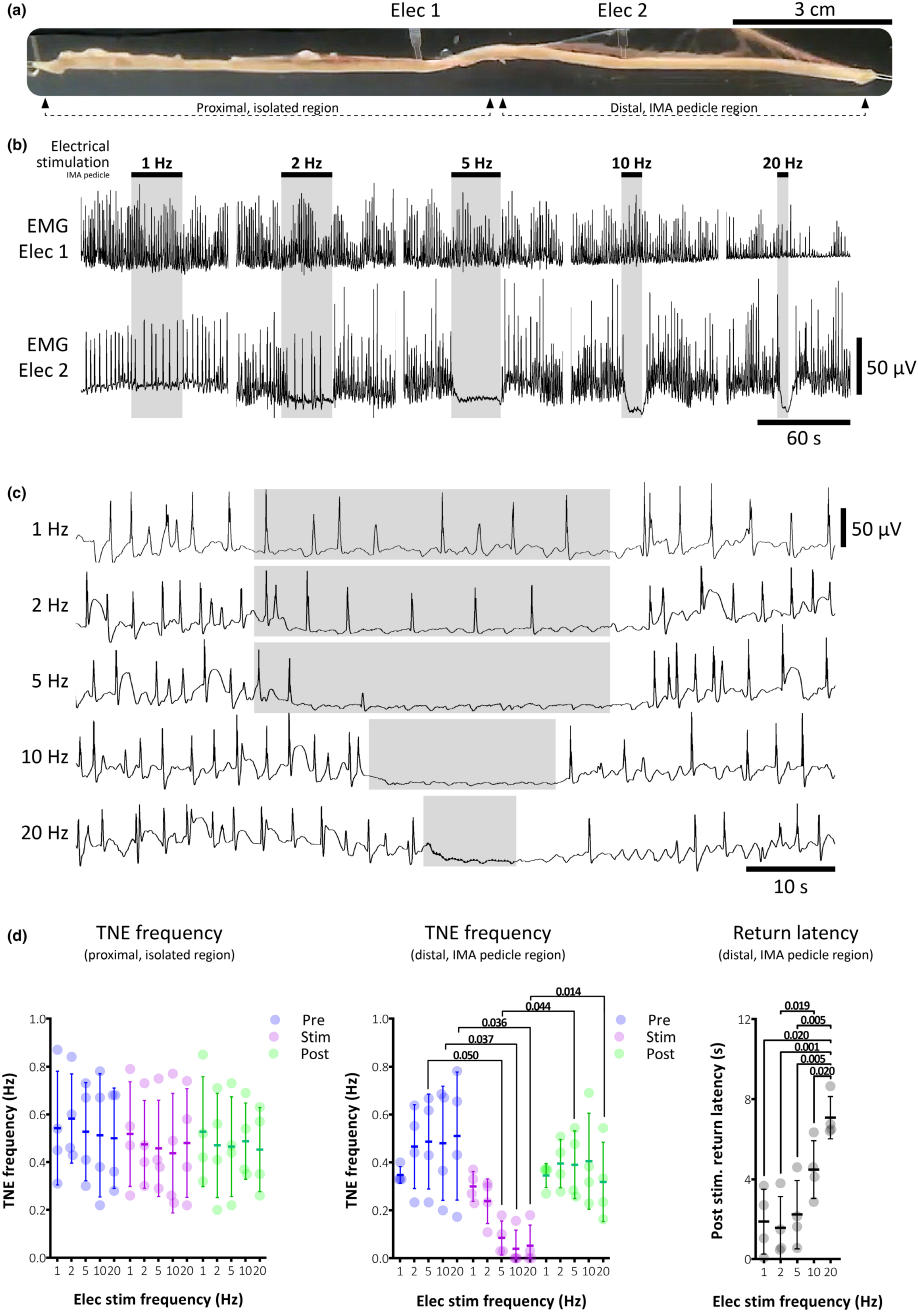
Inhibition of TNEs in guinea pig distal colon by sympathetic nerve stimulation. (a) Photograph of a preparation during recording of TNEs by dual suction electrodes. The proximal electrode 1 was located on the serosal surface of the gut wall in the extrinsically isolated region. Electrode 2 was placed in the IMA pedicle region, with intact extrinsic nerves. (b) Example series showing the effects of IMA pedicle stimulation at different frequencies on TNEs recorded at each electrode site. There was no obvious effect on smooth muscle electrical activity at the proximal electrode, outside the IMA pedicle region at any stimulation frequency tested. However, the distal electrode (electrode 2) shows clear suppression of TNEs that was graded with stimulation frequency. (c) Examples of IMA pedicle stimulation at increasing frequencies on the distal electrode in a separate preparation to that shown in (b). These examples shown on an expanded time scale more clearly show the graded stimulation responses. Shaded regions represent the period of stimulation. (d) Summary data showing TNE frequencies across stimulation periods and the average latencies to the return of TNEs after different frequencies of stimulation (*n* = 4). Graphs of TNE frequencies show data before (pre, blue markers), during (stim, magenta markers), and after (post, green markers) IMA pedicle stimulation in the isolated region (left graph) and pedicle region (right graph). TNEs were significantly inhibited by IMA pedicle stimulation in the distal but not proximal region at stimulation frequencies of 5–20 Hz. *p* values represent Dunnett's post‐test following two‐way repeated measures ANOVA. The return latency graph shows how the period of TNE quiescence beyond the cessation of stimulation was positively graded with stimulation frequency, with the highest frequency incurring the longest durations of electrical quiescence. *p* values represent Tukey post‐test following one‐way repeated measures ANOVA. Individual dots represent each replicate animal; overlying bars represent the mean ± *SD*.

TNEs were recorded by both electrodes in empty colon preparations as abrupt depolarizations or hyperpolarizations with an ongoing frequency ranging 0.2–0.8 Hz (Figure 2; *n* = 4; 4/4 male). Stimulation of the IMA pedicle at frequencies of 1, 2, 5, 10, and 20 Hz, caused a graded suppression of the ongoing TNEs in the distal colonic region supplied by the IMA pedicle, but not in the proximal region (*p* = 0.028 and 0.271, main effect of stimulation in the distal and proximal regions, respectively, two‐way ANOVA; Figure 2b). In the pedicle region the effects of stimulation were visually apparent from the lowest frequencies (Figure 2c), while stimulation at 20 Hz caused membrane hyperpolarization and an extended period of inhibition of TNEs beyond the cessation of the stimulus (Figure 2b,c), averaging 7.1 ± 1.1 s (Figure 2d). At the IMA pedicle region, reductions in the frequency of TNEs were statistically significant at stimulation frequencies of 5 Hz and greater (*p* = 0.050, 0.037, and 0.036 for 5, 10, and 20 Hz, respectively, compared to baseline, Dunnett's post‐test, two‐way ANOVA, *n* = 4; Figure 2d). These data suggest that sympathetic input to the distal colon readily inhibits TNEs.

### 3.2 Colonic motor complexes (CMCs)

Mechanical forces associated with CMCs were recorded in four preparations by intraluminal catheter (*n* = 4; 3/4 male). CMCs were periodic, high amplitude pressure events that could propagate in any direction (Figure 5a). These were ongoing at a frequency of 0.80 ± 0.05 cpm (*n* = 4), with most (88 ± 19%) propagating the full length of distal colon preparations. The direction of CMC propagation was mostly antegrade (60 ± 28%, *n* = 4), and a small proportion (5 ± 10%) were retrograde. Numerous CMCs were initiated at the oral and anal ends of the distal colon and collided mid‐way along the preparation (35 ± 25%).

Apparent regional differences in propagation direction prompted separate analyses of CMCs in the proximal and distal halves of preparations. Using the manometry recording, the proximal analysis included all CMCs present in the proximal half of the specimen, disregarding any distal extension for the direction of propagation analysis. CMCs recorded by the sensors in the distal half of the specimen were analyzed in the same way. In the proximal half, 95 ± 10% of CMCs were antegrade, 5 ± 10% were simultaneous, and none were retrograde (*n* = 4). The distal half was significantly more likely to have retrograde CMCs compared to the proximal half (*p* < 0.001, Chi‐square, standardized residual = 2, *n* = 4): 40 ± 43% were antegrade, 40 ± 28% were retrograde, and 20 ± 28% appeared simultaneous. CMCs initiated in the proximal or distal regions were similarly likely to propagate the full length of preparations (85 ± 19% vs. 90 ± 20%, respectively, *p* = 0.633, Chi‐square, *n* = 4). CMC frequency was also similar in the two regions (0.82 ± 0.06 vs. 0.78 ± 0.05 cpm, proximal vs. distal half, respectively; *p* = 0.060, paired *t*‐test, *n* = 4).

**FIGURE 3:**
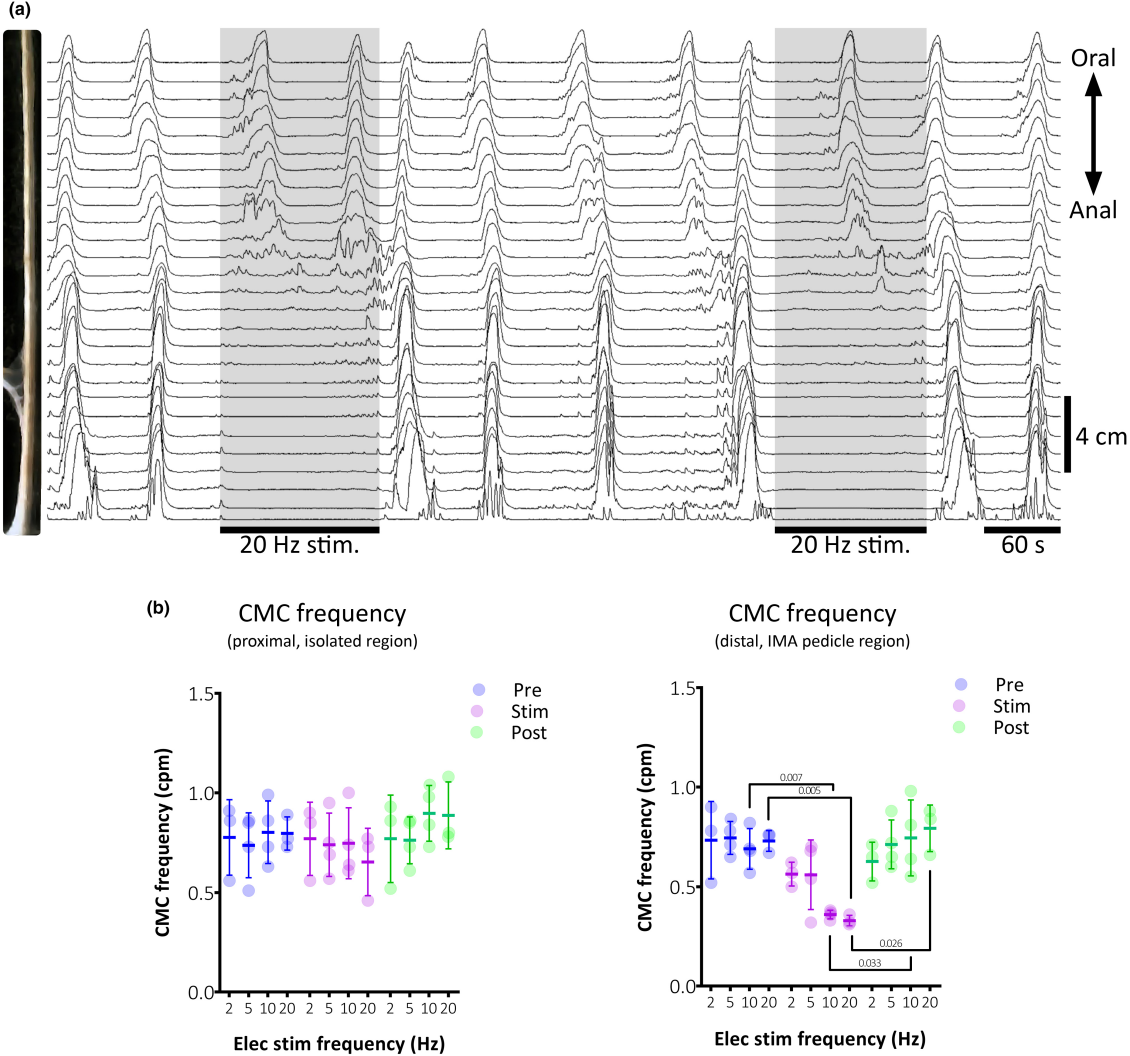
Effect of sympathetic nerve stimulation on CMCs in guinea pig distal colon. (a) Representative multi‐sensor recording of guinea pig distal colon showing ongoing CMCs and two instances of 20 Hz IMA pedicle stimulation for 120 s (shaded regions). Both stimulations abolished CMCs in the distal half of the preparation. During these periods, CMCs are not initiated in the distal segment. Additionally, CMCs persisted in the proximal segment at a normal frequency during stimulations but did not progress into the distal segment. (b) Summary data showing the significant reduction of CMC frequency by IMA pedicle stimulation at 10 and 20 Hz in the distal (right graph), but not proximal half of preparations (left graph; *n* = 4). *p* values represent Dunnett's post‐test following two‐way repeated measures ANOVA. Individual dots represent each replicate animal; overlying bars represent the mean ± *SD*.

**FIGURE 4:**
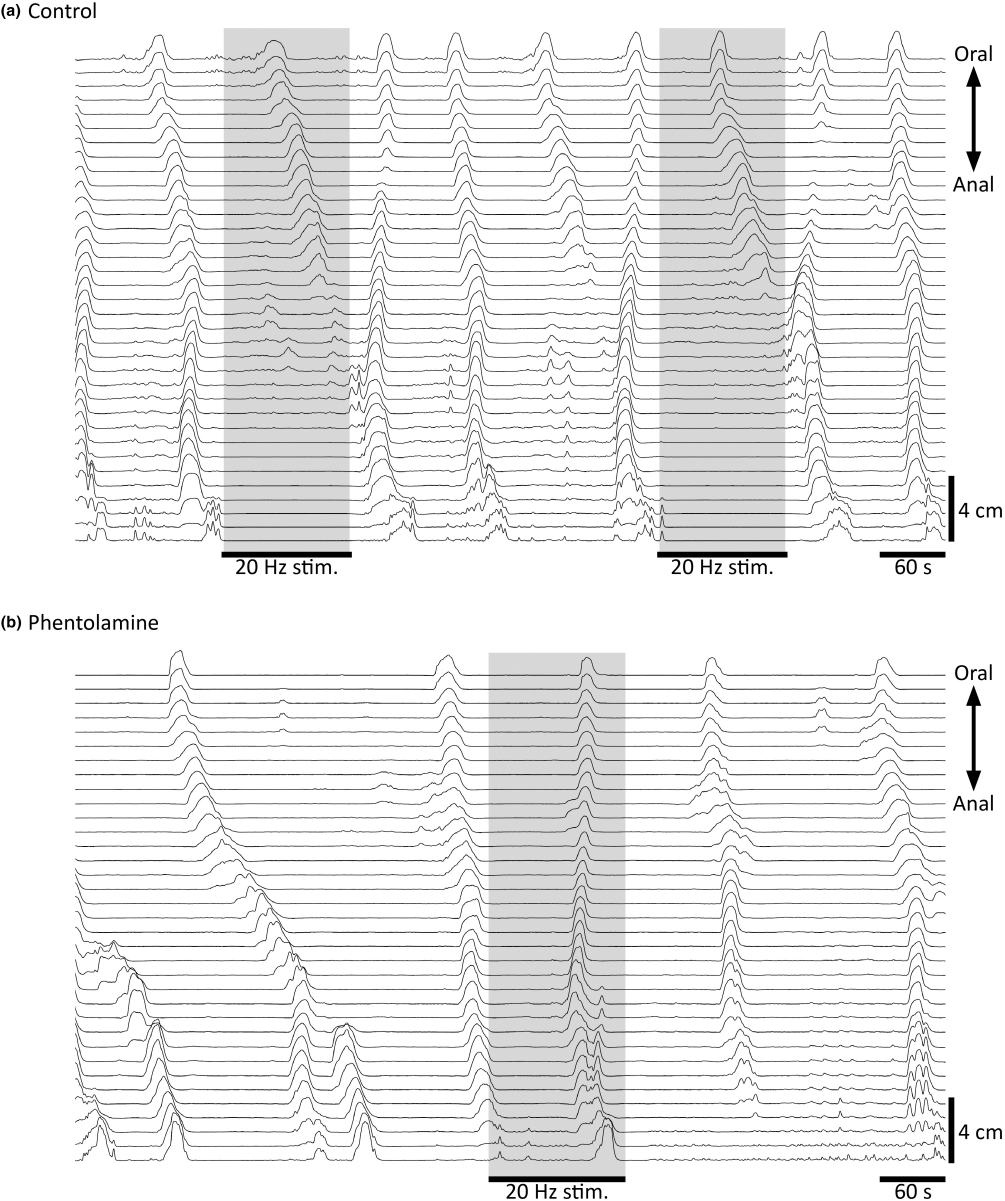
Sympatho‐inhibition of CMCs prevented by phentolamine. The non‐selective α adrenoreceptor antagonist, phentolamine (3.6 μM), was used in two preparations in which sympathetic nerve stimulation was tested on CMCs (*n* = 2). (a) Effects of 20 Hz IMA pedicle stimulations (120 s; shaded regions) on CMCs under control conditions and, (b) in phentolamine in the same guinea pig distal colon (*n* = 2). Phentolamine abolished the inhibitory effect of stimulation on CMCs seen in the distal half of the preparation where extrinsic inputs were intact.

During ongoing CMCs, colonic nerves following the IMA were electrically stimulated at frequencies of 2, 5, 10, and 20 Hz. In the distal portion of segments (with intact mesenteric nerves), stimulation at 2 and 5 Hz had no apparent effect, while 10 and 20 Hz caused significant interruption of ongoing CMCs (*p* = 0.002, main effect of stimulation, two‐way ANOVA, *n* = 4; Figure 3a,b). In contrast, CMCs persisted at a normal rate in the proximal region, at any stimulation frequency of the IMA pedicle (*p* = 0.169, main effect of stimulation, two‐way ANOVA, *n* = 4; Figure 3a,b). CMCs initiated in the proximal colon during 10 and 20 Hz stimulation, did not progress into the distal region. The inhibitory effect of 20 Hz stimulation was reversed by temporary removal of the IMA from the ring electrode, or by addition of phentolamine (see Figure 4a,b; *n* = 2/2 preparations tested; 3.6 μM).

CMCs in the proximal region maintained an antegrade preference during stimulation (68 ± 22%, 11 ± 15, and 6 ± 12%; antegrade, retrograde, and simultaneous, respectively; *n* = 4), as did those in the distal region (35 ± 26%, 10 ± 11, and 30 ± 33%; antegrade, retrograde, and simultaneous, respectively; *n* = 4). Thus, IMA pedicle stimulation at any frequency did not significantly affect CMC propagation direction in either the proximal or distal region of preparations (*p* = 0.184 and 0.515, proximal and distal regions, respectively, Chi‐square; *n* = 4). Taken together, these data suggest that sympathetic nerve stimulation requires higher frequencies to suppress CMCs than to abolish TNEs.

### 3.3 Pellet propulsion

**FIGURE 5:**
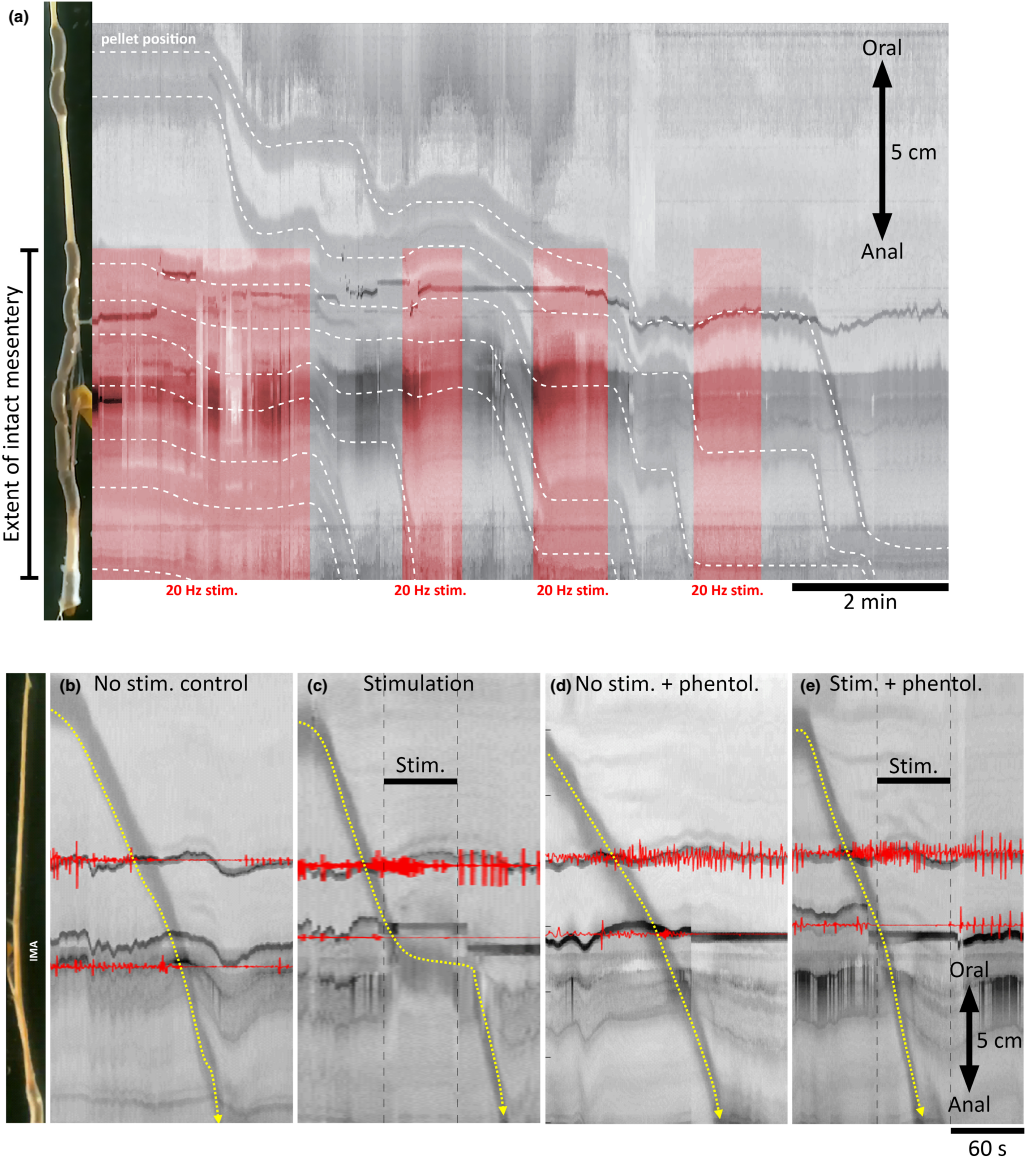
Effect of sympathetic nerve stimulation on pellet propulsion in guinea pig distal colon. Representative diameter maps (dmaps) showing the effect of stimulating the IMA pedicle on pellet propulsion (highlighted by dotted lines). (a) The progression of endogenous pellets during intermittent 20 Hz electrical stimulation of the IMA pedicle. Dashed white lines indicate the progress of each starting pellet over time. Note the tendency for moving pellets to be stopped and remain stationary during periods of stimulation and resume after cessation (*n* = 4). (b–e) In a separate series of experiments, the effects of sympathetic nerve stimulation on single pellet propulsion were tested in five preparations (*n* = 5). (b) Pellet movement along the distal colon under normal conditions. C IMA pedicle stimulation interrupts pellet movement, holding it largely stationary until cessation of stimulation, when it promptly resumes. (d) Example of pellet propulsion in phentolamine (3.6 μM), without IMA pedicle stimulation (tested in 2 of the 5 preparations). (e) IMA pedicle stimulation had no effect on pellet propulsion in the presence of phentolamine (2 of 2 preparations tested; *n* = 2), suggesting the inhibitory effects of IMA pedicle stimulation are mediated by α adrenoreceptors.

Three distal colon preparations containing 7–10 endogenous fecal pellets were video recorded during intermittent 20 Hz IMA pedicle stimulation (*n* = 4; 4/4 male). Stimulations typically arrested pellet propulsion, resuming on cessation of the stimulus (Figure 5a). Similar to TNEs, the inhibitory effect was apparently limited to the region immediately supplied by the IMA pedicle; pellet propulsion outside this region (more proximally) did not appear affected during stimulation, thus pellets clustered in the region of intact sympathetic innervation. An example of this is shown in Figure 5a. The buoyancy of clustered endogenous pellets caused movements of the gut toward the surface of the Krebs solution, making the experiment technically difficult to control. Nevertheless, in the absence of stimulation, endogenous pellets were expelled from preparations at an average rate of 1.02 ± 0.51 pellets per minute, which was reduced to 0.26 ± 0.33 pellets per minute during stimulation (*p* = 0.022, paired samples *t*‐test, *n* = 3). These data indicated sympathetic nerve stimulation inhibits pellet propulsion.

To compare the effectiveness of sympathetic stimulation on pellet propulsion to inhibition of TNEs and CMCs under more controlled conditions, 5 distal colon preparations were set up and single artificial fecal pellets were introduced to the oral end (*n* = 5). These pellets were allowed to be propelled to the anal end and expelled (Figure 5b). During pellet propulsion, the IMA pedicle was stimulated at frequencies of 2, 5, 10 or 20 Hz. At a frequency of 2 Hz, stimulation did not interrupt pellet propulsion in any instance (0/4 tested, *n* = 4). However, at 5 Hz stimulation, pellet propulsion was stopped in 3 of 5 tests (60%); in 4 of 5 tests at 10 Hz (75%); and in 5 of 5 tests at 20 Hz (adjusted standardized residual >2 for 5, 10, and 20 Hz, *p* = 0.020, Chi‐square; *n* = 4). After interruption of pellet propulsion by IMA pedicle stimulation, propulsion resumed following an average 15 ± 13 s latency from the cessation of stimulation (Figure 5c). Thus, the effect of sympathetic nerve stimulation on the propulsion of single pellets was statistically significant from a frequency of 5 Hz and greater and was similar to effects on TNEs. The addition of phentolamine (3.6 μM; see Figure 5b–e) abolished the effect of 20 Hz sympathetic nerve stimulation on pellet propulsion in 4 of 4 tests (*n* = 3). Figure 5b–e shows the effects of sympathetic nerve stimulation on pellet propulsion with and without the application phentolamine in the same preparation.

## 4 DISCUSSION

Transient neural events, CMCs and pellet propulsion are distinct neurogenic motor behaviors driven by different neural circuits and dynamics, raising the possibility of differential sensitivity to sympathetic input. The results of our experiments suggest that sympathetic inhibition affects colonic propulsion of single pellets and TNEs at lower stimulation frequencies compared to CMCs. This finding is compatible with the idea that colonic pellet retention may be facilitated by CMC‐activated peripheral sympathetic pathways which inhibit more effectively pellet propulsion than the CMCs themselves. Additionally, the persistence of TNEs and CMCs during effective sympathetic inhibition in adjacent, extrinsically denervated regions of colon, shows that stimulation of peripheral sympathetic pathways can exert spatially precise and fully reversible control of gut motility.

### 4.1 Source of visceromotor inhibition from colonic nerve stimulation

Prevertebral neurons represent ~80% of the sympathetic neurons to guinea pig distal colon, with the remaining 20% arising from paravertebral ganglia (Olsson et al., 2006). Prevertebral, but not paravertebral ganglia contain visceromotor neurons that directly inhibit gut motor circuits (Jänig & McLachlan, 1992). Prevertebral sympathetic axons in colonic nerve trunks that were stimulated in the present study are most likely to originate from the inferior mesenteric ganglia, but smaller contributions from the superior mesenteric and celiac ganglia also occur (Messenger et al., 1994). The myenteric plexus is the major target of sympathetic nerves in colonic nerve trunks, providing a high density of TH‐immunoreactive varicose fibers and pericellular baskets (Olsson et al., 2004; Tassicker et al., 1999). The inhibitory effects of sympathetic nerve firing within the guinea pig intestinal myenteric plexus are principally mediated by noradrenaline acting on presynaptic α_2_‐receptors on enteric neurons. This inhibits excitatory neurotransmission within the ENS by suppressing acetylcholine release (Hirst & McKirdy, 1974; Stebbing et al., 2001). In addition, α_1a_‐receptors have been implicated in colonic smooth muscle relaxation via PDGFRα+ cells in mouse and human (Kurahashi, Kito, Baker, et al., 2020; Kurahashi, Kito, Hara, et al., 2020). Other mechanisms, such as direct noradrenergic relaxation of smooth muscle (Beani et al., 1969; Furness, 1969; Gillespie, 1962), purinergic glial‐cell activation (Gulbransen et al., 2010), and activation of intrinsic inhibitory neurons (Zhang et al., 2022) are possible contributing mechanisms. That sympathetic inhibition of CMCs and pellet propulsion in the present study was blocked by phentolamine is compatible with a predominantly adrenergic mechanism via α receptors. However, sympathetic neurons are not the only source of axons in colonic nerve trunks. Peptide containing spinal afferent nerve fibers and axons of viscerofugal neurons are also present (Chen et al., 2015; Olsson et al., 2006; Tassicker et al., 1999) and were probably also activated by electrical stimulation in the present study, giving rise to antidromic action potentials. The lack of inhibition following phentolamine in the present study suggests these fibers contributed little to the inhibitory effects of colonic nerve stimulation. Indeed, there is potential for efferent‐like release of CGRP and tachykinins from spinal afferent neurons (Holzer, 2007), raising the possibility of excitatory effects on motility (Hibberd et al., 2022). However, optogenetic studies in mice indicate little effect of spinal afferent nerve activation in the absence of intact spinal cord circuits (Smith‐Edwards et al., 2019). Taken together, this suggests that the inhibitory effects of colonic nerve stimulation in the present study were most likely to be mediated by noradrenergic prevertebral sympathetic nerve fibers.

### 4.2 Stimulation of sympathetic pathways exerts localized inhibition

Sympathetic inhibition under all experimental conditions, regardless of stimulation frequency, showed containment to the region with intact extrinsic nerves to the IMA pedicle without generalizing along the entire distal colon. For example, TNE inhibition was evident only at the distal suction electrode (Figure 2a,b), and the inhibition of endogenous pellet movement did not appear effective unless pellets moved within the extrinsically intact region (Figure 4a). The most overt display of localization was shown by manometric recordings during the inhibition of CMCs (Figure 3a). Stimulation prevented both local CMC initiation and the invasion of CMCs initiated elsewhere into the extrinsically intact region, allowing CMC activity to persist at regular frequency outside this region (Figure 3b). This provides proof of principle that electrical neuromodulation approaches can be regionally constrained, which is of potential clinical interest since localized functional deficits can feature in gut motility disorders, including fecal incontinence (Lin et al., 2017).

### 4.3 Entero‐sympathetic circuits and motility control

Peripheral colo‐colonic reflex pathways that can inhibit motility were first demonstrated in the 1940 s (Kuntz, 1940; Kuntz & Saccomanno, 1944). The underlying neural circuit comprises enteric viscerofugal neurons in the afferent arm, and gut‐projecting postganglionic sympathetic prevertebral neurons in the efferent arm (Szurszewski et al., 2002). Viscerofugal neurons are activated by gut distension and input from other enteric neurons (Hibberd et al., 2012a; Miller & Szurszewski, 1997). Recordings from guinea pig viscerofugal neurons suggested they could be strongly driven by motor circuits (Hibberd et al., 2012b). In mice, the firing pattern of the myenteric plexus underlying CMCs (Spencer et al., 2018) also drives viscerofugal neurons and, in turn, sympathetic postganglionic neurons (Hibberd et al., 2020). Viscerofugal nerve terminals synapse on visceromotor and secretomotor sympathetic nerve cell bodies rather than vasoconstrictor neurons. Thus, the peripheral entero‐sympathetic circuits are structured for regulating gut motility and secretion. However, the effects of the CMC‐driven activation of sympathetic neurons on different motility behaviors remain unclear. The results of the present study suggest that the efferent output of the circuit (and of sympathetic activation more generally), is more likely to affect pellets propulsion than the CMCs that can activate the circuit. Indeed, recordings from the afferent and efferent arms of the circuit in mouse revealed potent activation by CMCs that did not interfere with their ongoing occurrence (Hibberd et al., 2020). That is, CMCs were not self‐extinguishing, consistent with the present study and with mechanical recordings comparing CMCs in mouse colon with and without intact peripheral sympathetic circuits (Smith‐Edwards et al., 2020). This raises the possibility that ongoing CMC‐driven sympathetic activity could inhibit pellet propulsion without inhibiting the CMCs themselves to facilitate pellet retention in the colon. Further studies of the effects of the intact peripheral sympathetic circuits are needed to determine the validity of the hypothesis that they contribute to the capability of the guinea pig distal colon to accumulate pellets, in vivo.

## AUTHOR CONTRIBUTIONS

DJS, PGD, TJH, and MC performed experiments. DJS, MC, and TJH analyzed the data. All authors contributed to study design and interpretation. DJS, MC, PGD, and TJH wrote the manuscript. All authors edited and approved the final version of the manuscript.

## FUNDING INFORMATION

DJS had financial support from Medtronic for the duration of this project. Experiments in this study were supported by National Health and Medical Research Council (NHMRC) Project grants #1156416 and #1127140 to NJS and an Australian Research Council (ARC) Discovery Project grant #DP190103628 to NJS.

## CONFLICT OF INTEREST

The authors have no conflict of interest to declare.
